# Pollen Grain Preservation and Fertility in Valuable Commercial Rose Cultivars

**DOI:** 10.3390/plants6020017

**Published:** 2017-04-24

**Authors:** Annalisa Giovannini, Anca Macovei, Matteo Caser, Andrea Mansuino, Gian Guido Ghione, Marco Savona, Daniela Carbonera, Valentina Scariot, Alma Balestrazzi

**Affiliations:** 1 Council for Agricultural Research and Economics (CREA), Ornamental Species Research Unit, Corso degli Inglesi 508, 18038 Sanremo (IM), Italy; marco.savona@crea.gov.it; 2 Department of Biology and Biotechnology “Lazzaro Spallanzani” (DBB), via Ferrata 9, 27100 Pavia, Italy; anca.macovei@unipv.it (A.M.); daniela.carbonera@unipv.it (D.C.); alma.balestrazzi@unipv.it (A.B.); 3 Department of Agricultural, Forest and Food Sciences, University of Turin, Largo Paolo Braccini 2, 10095 Grugliasco (TO), Italy; matteo.caser@unito.it (M.C.); valentina.scariot@unito.it (V.S.); 4 NIRP International, Az. Agricola di Ghione L. & Figli, via San Rocco 1, Fraz. Bevera, 18039 Ventimiglia (IM), Italy; andream@nirpinternational.com (A.M.); gianguidog@nirpinternational.com (G.G.G.)

**Keywords:** anther conservation, frozen pollen, in vitro germination, pollen tube growth

## Abstract

In the cut flower market, traditional breeding is still the best way to achieve new rose cultivars. The geographical delocalization of cultivar constitution (generally made in Europe and North America) and plant cultivation (large areas in Africa and South America) represents a limit point for crossing and selection. Rose breeders often need to overcome geographical distances, resulting in asynchrony in flowering among crossing parents, by storing and sending pollen. Hence, a key aspect in breeding programs is linked to pollen availability and conservation, jointly with the identification of parameters related to pollen fertility. In this study we present the results of three different trials. In the first, pollen diameter and pollen viability were chosen as fertility predictors of 10 *Rosa hybrida* commercial cultivars. In the second trial, aliquots of dried pollen grains of six *R. hybrida* cultivar were stored under two different temperatures (freezer at T = −20 °C and deep freezer at T = −80 °C) and after a wide range of conservation period, their viability was measured. In the third trial, the effective fertilization capacity of frozen pollen of 19 pollen donor cultivars was evaluated during 2015 crossing breeding plan, performing 44 hybridizations and correlating the number of seeds and the ratio seeds/crossing, obtained by each cultivar, with in vitro pollen germination ability.

## 1. Introduction

In the cut flower market, many modern rose cultivars are tetraploid (2n = 4x = 28) and they are produced by traditional genetic improvement methods (i.e., crossing and selection). Fertility varies considerably among rose cultivars and has led breeders to greatly prefer the more fertile genotypes as parents [1]. Rose breeding companies may overcome geographic distances and differences in flowering time by storing selected pollen until pollination of the flowers used as female parents can be performed. Pollen viability is genotype dependent and affected by the way of storage [2,3,4,5,6,7]. In fact, pollen longevity quickly decreases when maintained at ambient temperature and 50% humidity [8,9]. Although pollen germination rate tends to decline over storage, Zlesak et al. (2007) found that the pollen tube length of pollen stored for 2 or 52 weeks at −80 °C was comparable to fresh pollen and was significantly longer than that of pollen stored at −20 °C or 4 °C, pointing to deep freezing as a favourable temperature for general pollen storage [10]. Pollen degradation during storage conditions could be due to dehydration, which results in loss of pollen colloidal properties. In addition, reactive oxygen species (ROS) and reactive nitrogen species (RNS) over-accumulation can inhibit rose pollen germination. Recent studies on hybrid tea roses shows that the viable level of fresh pollen varies among cultivars and also the pollen preservation at −20 °C and −80 °C is cultivar dependent [11].

In the present study, pollen diameter and viability were related to fertility in *Rosa hybrida* L. commercial cultivars. Furthermore, an efficient protocol for pollen storage under low temperatures was applied to six cultivars in order to establish pollen preservation procedures useful for rose breeding and germplasm resource research. The effective fertilization capacity of the preserved pollen was studied in 2015 breeding plan of the company NIRP International (Italy) and the correlation between pollen viability and fertility was investigated.

## 2. Experimental Section

### 2.1. Pollen Diameter

Anthers were collected from *Rosa hybrida* “Alba”, “Aubade”, “Clipper”, “Encanto”, “Green Fashion”, “Golden Fashion”, “Marvelle”, “Peach Aubade”, “Variance”, and “Whisper” plants cultivated in the NIRP International greenhouse (Bevera, IM, Northwest Italy). Anthers were air dried for 24 h at standard conditions (T = 24 °C) to favour release and drying of the pollen [7]. A total of 100 mg of dry pollen grains were dusted onto a glass slide without a cover slip. The mean diameter of the normal pollen was calculated for each genotype, as well as its percentage over the whole population. The longest axis was measured through microscopic observations under a Leica DMIRB microscope (Leica Microsystems GmbH, Wetzlar, Germany). Subsequently, one drop of a medium composed of 10% sucrose *w*/*v*, 0.01% H_3_BO_3_, 0.01% CaCl_2_, 0.02% MgSO_4_·7H_2_O, 0.01% KH_3_PO_4_ and 0.01% chloramphenicol was added to the glass slide and new measurements were performed after 5 min of hydration. Pollen diameter was measured digitally using software LAS software (Leica Application Suite). Since the abnormal pollen maintains irregular shape after hydration [4], to define the frequency of normal pollen, the number of spherical pollen grains were counted.

### 2.2. Storage at Low Temperatures

**Figure 1:**
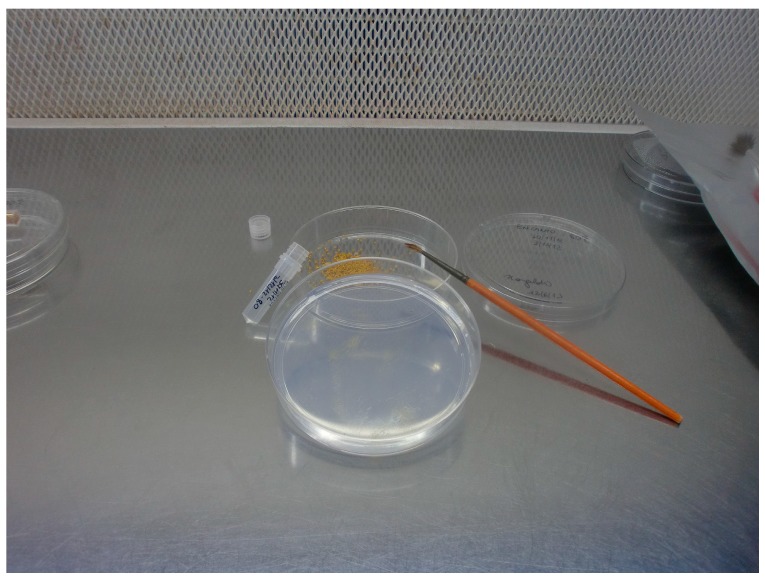
Example of rose anthers stored at low temperatures for conservation trials. Over the storage period, germination readings were made by immediately spreading pollen with a paint brush on a Petri dish filled with the culture medium. Reproduced from Giovannini et al. (2015) [6], with permission of ISHS.

Flowers of six *Rosa hybrida* “Alba”, “Anastasia”, “Encanto”, “Marvelle”, “Swan” and “Touch of Class” were collected at bud starting blooming stage, from plants cultivated in the NIRP greenhouses. The pollen grains were obtained from a bulk of twelve flowers by gathering the anthers during November 2012. Anthers were air dried for 24 h at room temperature (T = 24 °C 50% humidity) to favour release and drying of the pollen, then they were weighted in aliquots, of 100 mg and stored in polyethylene tubes at T = −20 °C in the freezer and at T = −80 °C in the deep freezer (Figure 1). The percentage of pollen germination (mean ± standard error) was evaluated on artificial culture medium [4] after 24 h at 24 °C, (Day 0), in dark conditions, and after a cold storage period of 44, 134 and 190 days, respectively [6]. In a preliminary test we have observed that pollen germination of control was reduced to zero after 10 days at 24 °C (data not shown). Pollen was immediately placed on the germination medium, under the laminar flow. When the pollen tube length was one and a half the diameter, the pollen grain was considered germinated. The observations were done on four replicates of 160 samples (640 normal pollen grains with a diameter larger than 30 μm), for each storage time and temperature, for each cultivar.

### 2.3. Effective Fruiting Using Preserved Pollen

Flowers of 19 *Rosa hybrida* commercial cultivars “Alba”, “Asante”, “Avalon”, “Dallas”, “Freedom”, “Golden Fashion”, “Grande Amore”, “High’n Magic”, “Mohana”, “Mondial”, “Nordia”, “Rafiki”, “Samourai”, “Sonrisa”, “Stardust”, “Swan”, “Tropical Amazone”, “Upper Class” and “Upper Gold” were collected in summer 2014, and pollen was coldstored using the protocol described above. Aliquots of dried anthers (100 mg) were stored in plastic boxes in the freezer (T = −20 °C) for a year. Pollen viability was tested in April 2015 by measuring the in vitro germination percentages of 160 samples for each cultivar on the artificial germination medium described above. Aliquots of the same pollen were used as donor in 44 hybridizations listed in Table 1. Stored pollen, in the freezer up to 12 months, of 19 commercial cultivars, was used in 2015 breeding program of the NIRP International Company. Forty-four hybridizations were performed during summer. The crossing quantity (number of seeds produced) was counted for each crossing.

### 2.4. Statistical Analysis

Arcsine transformation was performed on all percent incidence data before statistical analysis in order to improve homogeneity of variance. Effects of genotype and storage duration on the analysed traits were evaluated by one-way ANOVA using Ryan-Einot-Gabriel-Welsch’s multiple stepdown F (REGW-F) test (*p* ≤ 0.05). Pearson correlation index was calculated between pollen viability (in vitro germination %), the number of seeds and the ratio seeds/crossing. All analyses were performed with SPSS 21.0 Inc. software (Chicago, IL, USA).

## 3. Results

**Figure 2:**
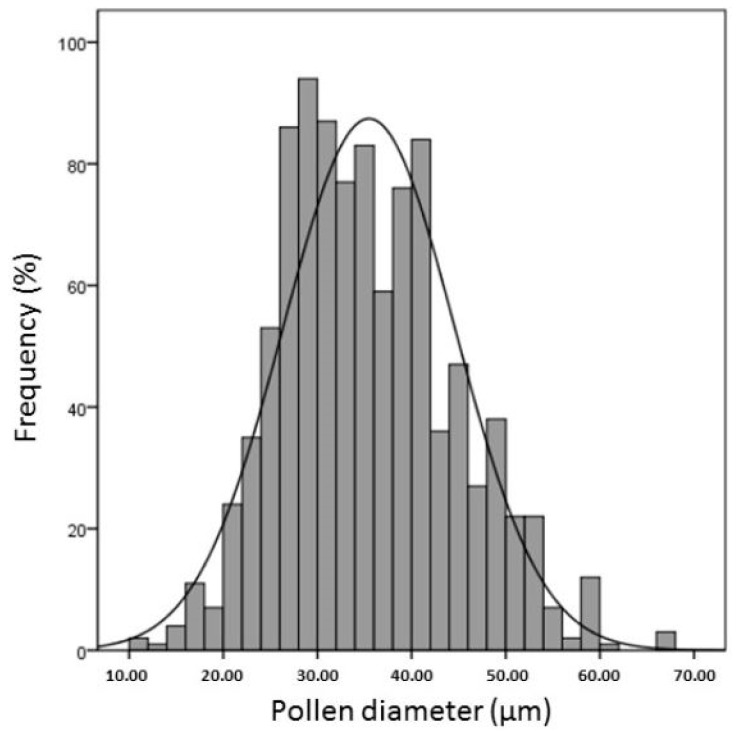
Frequency distribution of the pollen diameters (µm) of the 10 studied rose cultivars measured on the whole population.

In *Rosa hybrid* commercial cultivars, the mean dry pollen diameter ranged from 11.62 µm to 67.16 µm, (Figure 2) with an average of 35.47 µm. Among genotypes (Figure 2), “Clipper” showed the lowest mean diameter with 28.7 ± 4.10 µm, while the highest was “Green Fashion” (41.6 ± 8.02 µm).

**Figure 3:**
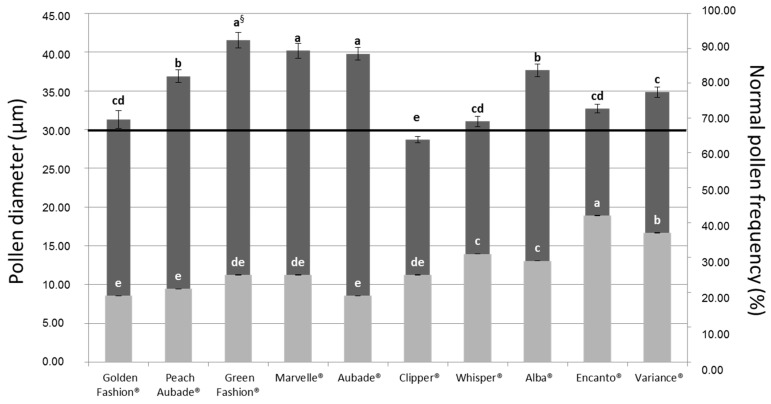
The pollen diameter (µm) (dark grey histograms) and the frequency of normal pollen (%) (light grey histograms) of the 10 studied rose cultivars. The black line at 30 µm represents a threshold between low fertile pollen (<30 µm) and high fertile pollen (>30 µm) [4]. ^§^ Letters indicate the significance of differences between genotypes as determined by one-way ANOVA and Ryan-Einot-Gabriel-Welsch’s multiple stepdown F (REGW-F) post-hoc tests. Vertical bars indicate ± standard error.

After hydration, microscopic observations revealed two types of pollen grains: undeveloped with a diameter smaller than 30 µm (abnormal) and globular with a diameter larger than 30 µm (normal) [4]. The frequency of normal pollen ranged between 18.8 ± 2.0% (“Aubade”) and 42.3 ± 8.0% (“Encanto”) (Figure 3). In this study, only one of the 10 analysed commercial cultivars showed diameter lower than 30 µm (“Clipper”).

**Figure 4:**
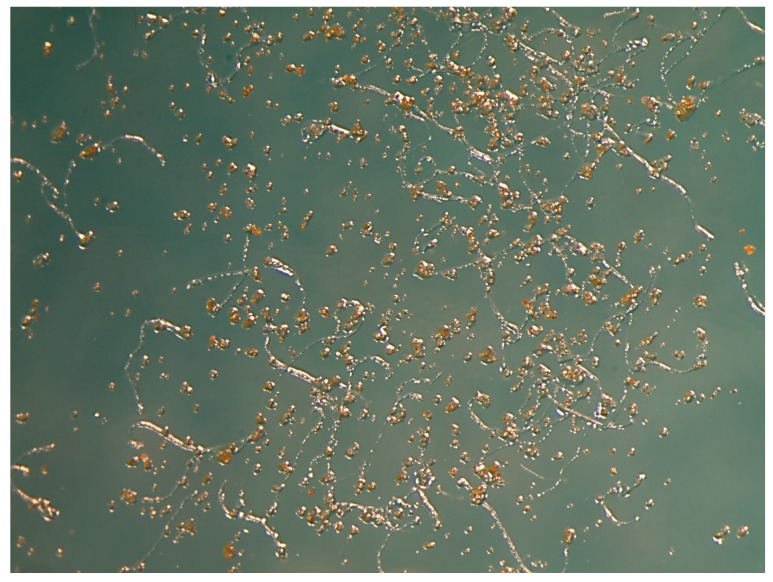
“Encanto” rose pollen spread on the culture medium, containing 40 mg L^−1^ H_3_BO_3_; 152 mg L^−1^ CaCl_2_(H_2_O); 150 g L^−1^ sucrose; 7 g L ^−1^ agar with a pH of 5.6, after 190 days of storage in the deep freezer (T = −80 °C). Pollen tubes developed from the germinated pollen grains.

About one gram of anthers was recovered from each cultivar (“Alba” 0.914 g, “Anastasia” 1.115 g, “Encanto” 1.353 g, “Marvelle” 1.079 g, “Swan” 0.774 g and “Touch of Class” 1.031 g). A cultivar-dependent behaviour in fresh pollen germination efficiency was observed. The cultivars “Anastasia” and “Marvelle” showed very low in vitro germination rates (0.985 ± 0.47 and 0.875 ± 0.38%, respectively), soon after flower collection (Day 0). Their pollen grains were not further used in the conservation trials. The best performing cultivars at Day 0 were “Alba” and “Encanto” with 57.1 ± 3.04 and 55.1 ± 1.34% pollen germination, respectively. After 44 days of storage at low temperatures, pollen germination was reduced in the cultivars “Swan” and “Touch of Class”, while no significant differences were observed in “Encanto”. After 134 days of storage pollen germination capacity was also reduced in the cultivar “Alba”. The cultivar “Encanto” with the highest frequency of normal pollen, increased pollen germination after 134 and 190 days of storage both in the freezer and in the deep freezer (Figure 4). Pollen in vitro germination was checked before the breeding season and it ranged from low (6% in “Dallas” and “Mohana”) to very high (99% in “Avalon” and “Stardust”), depending on the cultivar. Table 1 shows the number of crossings, the number of seeds and the ratio seed/crossing obtained by each cultivar. There was a statistically positive correlation (Pearson correlation coefficient *ρ* = 0.343 and *ρ* = 0.304) between stored pollen in vitro germination and in vivo fertility of the same pollen (Table 2).

## 4. Discussion

The performance of rose pollen has been studied by several authors [1,2,4,12,13]. A review of current knowledge of rose pollen morphology, formation, release, management, germination and fertilization has been recently published [7]. In Hybrid roses the pollen is a fine yellow colour dust, which is released by the anthers depending by ambient air, temperature and solar radiation. Rose pollen is naturally adapted to survive in arid environments. The shape is elliptical and furrowed by three longitudinal pores from which the pollen tube emerges during the fertilization process [7].

In Hybrid roses, a few reliable models for fertility prediction have been published. Pipino et al. (2011) reported on the correlation which associates pollen morphology with the number of seeds produced per hybridization. A diameter value of 30 μm was identified by the authors as the threshold between high and low pollen fertility [1,14]. In this study, only one out of the 14 tested commercial *Rosa hybrida* cultivars showed diameter lower than 30 μm (“Clipper”) and low fertility could be associated also to low viability and germinability percentages.

**Figure 5:**
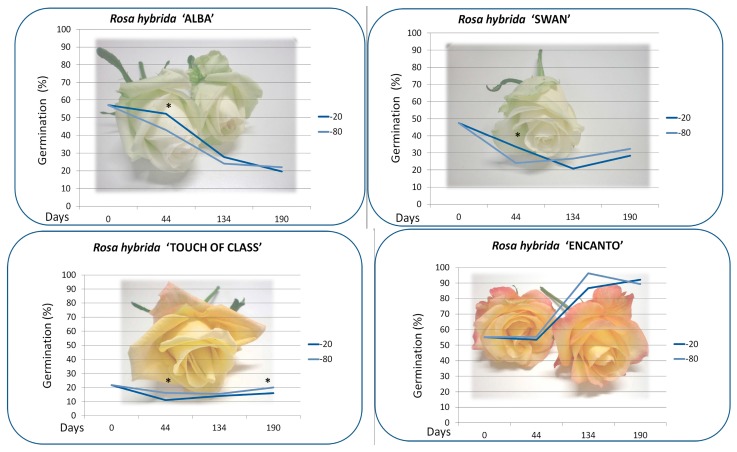
“Alba”, “Swan”, “Touch of Class” and, “Encanto” rose pollen germination percentages at Day 0 (fresh pollen) and after 44, 134 and 190 days of storage at −20 °C and −80 °C. For each experimental point the asterisk indicates significant differences by one-way ANOVA at 5% probability. Reproduced from Giovannini et al. (2015) [6] with permission of ISHS. The germination percentage of the untreated pollen control stored at room temperature was reduced to zero within 10 days (data not shown).

According to our previous observations, the viable level of fresh rose pollen is cultivar dependent and related to the temperature of conservation [6]. At room temperature rose pollen germination quickly decreases. The effect of two storage temperatures (−20 °C and −80 °C) was compared for the commercial rose cultivars “Alba”, “Encanto”, “Swan” and “Touch of Class” and few differences were observed between them (Figure 5). The conservation of genetic resources at low temperatures is a cost-effective technique for vegetative propagated crops [2]. The protracted storage time increases the portion of pollen grains able to germinate for nine wild rose species that showed viability higher or similar to that before storage at −25 °C for six months [12]. The kiwi pollen grains of the cultivar “Tomuri”, stored in the refrigerator or in the freezer, can be used for pollination in the same growth cycle [15].

Our in vivo hybridization results endorsed the positive correlation between stored pollen in vitro germination and in vivo fertility.

## 5. Conclusions

The morphology (mean dry pollen diameter) and the frequency of normal pollen (30 µm) are effective as fertility predictors in *Rosa hybrida* commercial cultivars.

Confirming the pioneer experiments of Calvino Mameli E. (1951) [8], rose pollen germination is cultivar dependent and can be achieved on artificial media containing, among other things, sugar and boric acid. Moreover, pollen can be stored at low temperatures, without resetting the original viability. The study on effective fruiting using one year frozen pollen reveals that there is a positive correlation between in vitro pollen germinability and seed set. The fertility capacity of the pollen donor cultivars is maintained after the storage conditions. Our results validate that preserved pollen at low temperatures is a useful tool for breeders and can be routinely used in automated breeding systems.

## Figures and Tables

**Table 1:** Hybridizations performed in 2015 NIRP International breeding plan. Rose maternal plant and pollen donor cultivars are list in the table; the number of crossings made in 2015 breeding plan, the number of collected seeds and the ratio seed/crossing are reported. Pollen in vitro germination percentages of the pollen donor cultivars are in the last column on the right letters indicate the significance of differences between genotypes as determined by one-way ANOVA and REGW-F post-hoc tests.

Maternal Plant Cultivar	Pollen Donor Cultivar	Crossing Number	Seed Number	Seed/Crossing	Pollen Donor In Vitro Germination
“Stardust”	“Alba”	15	2 h	0.13 e	26% e
“Red Naomi”	“Asante”	73	0 h	0 e	24% e
“Furiosa”	“Asante”	135	0 h	0 e	24% e
“Movie Star”	“Avalon”	62	537 a	8.66 a	99% a
“Sonrisa”	“Avalon”	112	192 cd	1.71 c	99% a
“Red Serenade”	“Dallas”	18	34 f	1.8 c	6% f
“Star Rose”	“Freedom”	37	1 h	0.02 e	98% a
“Grande Classe”	“Freedom”	82	0 h	0 e	98% a
“Hot Party”	“Freedom”	42	78 e	1.85 c	98% a
“Dejavù”	“Golden Fashion”	95	0 h	0 e	22% e
“Mohana”	“Golden Fashion”	85	0 h	0 e	22% e
“Grand Prix”	“Grande Amore”	56	41 f	0.73 d	77% c
“Encanto”	“High’n Magic”	22	35 f	1.59 c	97% a
“Jupiter”	“High’n Magic”	86	153 d	1.77 c	97% a
“Red Calypso”	“High’n Magic”	123	7 gh	0.05 e	97% a
“Havana”	“Mohana”	13	10 gh	0.76 d	6% f
“Dejavù”	“Mohana”	21	0 h	0 e	6% f
“Stardust”	“Mohana”	16	25 fg	1.56 c	6% f
“Polar Star”	“Mondial”	106	167 d	1.57 c	98% a
“Carioca”	“Nordia”	33	21 fg	0.63 d	50% d
“Variance”	“Nordia”	55	37 f	0.67 d	50% d
“White Angel”	“Nordia”	10	6 h	0.60 d	50% d
“Avalanche”	“Nordia”	88	3 h	0.03 e	50% d
“Jupiter”	“Rafiki”	98	267 bc	2.72 c	96% a
“Tropical Delight”	“Rafiki”	72	2 h	0.02 e	96% a
“Red Calypso”	“Rafiki”	134	123 d	0.91 d	96% a
“Vegas”	“Samourai”	134	0 h	0 e	8% f
“Grand Prix”	“Samourai”	15	0 h	0 e	8% f
“Upper Class”	“Samourai”	115	22 fg	0.19 de	8% f
“Opera”	“Samourai”	103	10 gh	0.09 e	8% f
“Upper Gold”	“Sonrisa”	89	0 h	0 e	68% cd
“Sonrisa”	“Stardust”	100	237 c	2.37 c	99% a
“Swan”	“Stardust”	54	220 c	4.07 bc	99% a
“Tara”	“Stardust”	97	31 f	0.31 de	99% a
“Mohana”	“Stardust”	12	0 h	0 e	99% a
“Polar Star”	“Swan”	94	17 g	0.18 de	39% e
“Fox Trot”	“Tropical Amazone”	113	571 a	5.05 b	63% cd
“Movie Star”	“Tropical Amazone”	76	456 b	6 b	63% cd
“Avant Garde”	“Upper Class”	102	588 a^§^	5.76 b	88% b
“Miss Paris”	“Upper Class”	19	5 h	0.26 de	88% b
“Grand Prix”	“Upper Class”	22	0 h	0 e	88% b
“Burgundy”	“Upper Class”	44	2 h	0.04 e	88% b
“Furiosa”	“Upper Class”	102	71 e	0.69 d	88% b
“Dejavù”	“Upper Gold”	15	0 h	0 e	32% e

**Table 2:** Correlations between in vitro germination of preserved pollen in *Rosa hybrida* commercial cultivars and the fertility parameters (number of seeds and the ratio seeds/crossing), in modern rose commercial cultivars.

	Pollen Germination (%)	Seed Number	Seed/Crossing
Pollen germination (%)	1		
Seed number	0.343 *	1	
Seed/crossing	0.304 *	0.938 **	1

* Correlation (Pearson) significant = *p* < 0.05 (2-tailed). ** Correlation (Pearson) significant = *p* < 0.001 (2-tailed).
